# Case Report: Pediatric nephrology—expanding the genotypic spectrum of *COQ2*-related nephropathy with a novel splice site variant in CoQ10-responsive SRNS

**DOI:** 10.3389/fmed.2026.1682564

**Published:** 2026-02-12

**Authors:** Yu-Ren Huang, Abhijeet Pal, Anne Chun-Hui Tsai

**Affiliations:** 1 Department of Genetics and Genome Sciences, Case Western Reserve University School of Medicine and University Hospitals, Cleveland, OH, United States; 2 Department of Urology, Case Western Reserve University School of Medicine and University Hospitals, Cleveland, OH, United States; 3 School of Medicine, China Medical University College of Medicine, Taichung City, Taiwan; 4 Section of Nephrology, Department of Pediatrics, St. Christopher's Hospital for Children, Philadelphia, PA, United States; 5 Section of Genetics, Department of Pediatrics, University of Illinois Chicago, Chicago, IL, United States

**Keywords:** *COQ2*, nephrotic syndrome, ubiquinone, genetics, case report

## Abstract

Coenzyme Q10, also known as CoQ10, CoQ, and ubiquinone is an essential component of the mitochondrial electron-transport chain and functions as an energy transfer molecule as well as a redox carrier and is a lipid-soluble antioxidant. Biallelic pathogenic variants in one of the 10 genes encoding proteins involved in its synthesis, establishes the diagnosis of primary CoQ10 deficiency. *COQ2*, or parahydroxybenzoate-polyprenyltransferase (EC 2.5.1.39), catalyzes one of the final reactions in the biosynthesis of CoQ, the prenylation of parahydroxybenzoate with an all-trans polyprenyl group. *COQ2* related CoQ10 deficiency can present with multiple system atrophy, cardiomyopathy and steroid resistant nephrotic syndrome (SRNS). Multiple papers have suggested CoQ supplement can treat SRNS. We report a 9-year-old girl presenting with steroid-resistant nephrotic syndrome, whose renal biopsy revealed focal segmental glomerulosclerosis. She showed only a partial response to combined therapy with tacrolimus, lisinopril, and losartan. Whole exome sequencing identified two compound heterozygous variants in the *COQ2* gene (NM_015697.7): a known pathogenic variant, c.683A>G, inherited from her father, and a novel splice-site variant, c.692+3A>G, inherited from her mother and currently classified as a variant of uncertain significance (VUS). Notably, previously reported patients carrying the c.683A>G variant typically present with early-onset, severe disease. In contrast, our patient’s relatively late onset and isolated nephropathy suggests that the novel variant may be pathogenic but associated with a milder phenotype. Prompt genetic diagnosis enabled early initiation of high-dose CoQ10 supplementation (ubiquinone, 30 mg/kg/day), which led to marked clinical improvement and may have prevented further renal function deterioration or the development of other systemic manifestations.

## Introduction

Coenzyme Q10, also known as CoQ10, CoQ, and ubiquinone is an essential component of the mitochondrial electron-transport chain (ETC), a process that generates energy in the form of ATP (1, 2). It functions as both an energy transfer molecule and a redox carrier, and also acts as a lipid-soluble antioxidant (3). After its synthesis, CoQ is transported to the inner mitochondrial membrane, where it facilitates electron transfer between Complex II and Complex III of the ETC. Additionally, CoQ serves as an antioxidant within both intra-mitochondrial and extra-mitochondrial membranes, and plays a role in the regeneration of other antioxidants, including vitamins C and E (4).

The isoprenoid tail length of coenzyme Q varies by organism: humans essentially produce CoQ10, with only trace/uncertain CoQ9 likely attributable to diet or microbiota (5–7); rodents (e.g., mice) produce both CoQ9 and CoQ10, with CoQ9 predominant (8); *E. coli* produce CoQ8 (9), and *S. cerevisiae* produce CoQ6 (10). CoQ biosynthesis is governed by multiple genes, including *COQ2*, *COQ4*, *COQ5*, *COQ6*, *COQ7*, *COQ8A (ADCK3)*, *COQ8B (ADCK4)*, *COQ9*, *PDSS1*, and *PDSS2* (11, 12).

Coenzyme Q10 (CoQ10) deficiency can affect various organs, particularly those with high energy demands such as the brain, skeletal muscle, heart, eyes, and kidneys. Clinically, it presents with at least five major phenotypes: encephalomyopathy, severe infantile multisystemic disease, nephropathy, cerebellar ataxia with cerebellar atrophy, and isolated myopathy (13). Nephropathy typically manifests as steroid-resistant nephrotic syndrome (SRNS) and progressive renal dysfunction. Isolated myopathy, though less common, is an increasingly recognized presentation, often associated with secondary CoQ10 deficiency due to pathogenic variants in genes such as *ETFDH*, *ETFA*, and *ETFB*. These patients commonly exhibit proximal muscle weakness, elevated serum creatine kinase, and mitochondrial abnormalities on muscle biopsy, and they often respond favorably to CoQ10 and riboflavin supplementation (14–16). In contrast, primary CoQ10 biosynthetic gene defects (e.g., *COQ2, COQ4, COQ6*) more commonly result in multisystem involvement, though rare cases with muscle-predominant symptoms have also been reported (17).

Nephrotic syndrome is one of the most common glomerular diseases in pediatric populations. Approximately 10%–15% of affected individuals exhibit steroid-resistant nephrotic syndrome (SRNS), defined as persistent proteinuria unresponsive to corticosteroid therapy within 4–6 weeks (18). About 50% of SRNS patients respond to intensified immunosuppressive therapy, while the remainder develop multidrug resistance, progressing to chronic kidney disease (CKD) and ultimately to kidney failure (19). Notably, genetic mutations account for approximately 30% of childhood-onset SRNS cases and 10%–15% of adult-onset cases (19). Among these, pathogenic variants in the *COQ2* gene have been identified as a cause of CoQ10 deficiency-associated SRNS.

We present a case of SRNS caused by *COQ2* related CoQ10 deficiency, in which the patient showed clinical improvement following treatment with CoQ10 supplementation (ubiquinone).

## Case report

A 9-year-old girl was initially evaluated by nephrology services due to a 3-day history of periorbital swelling and generalized body edema. Her past medical history was notable for alopecia areata and asthma. Initial evaluation revealed periorbital edema and 2+ pitting edema in the lower extremities. Laboratory findings showed a BUN of 8 mg/dL, serum creatinine 0.41 mg/dL, and hypoalbuminemia with a serum albumin level of 1.6 g/dL. Urinalysis demonstrated 3+ proteinuria, and the urine protein-to-creatinine ratio (UPCR) was markedly elevated at 18.3 mg/mg.

Based on a presumptive diagnosis of nephrotic syndrome, high-dose oral prednisone (60 mg daily). However, due to persistent edema and inadequate outpatient response, she was admitted for albumin and furosemide (Lasix) infusions. She was discharged on furosemide 40 mg twice daily and prednisone.

After 8 weeks of high-dose corticosteroid therapy, she showed no clinical improvement, with persistent edema and ongoing 3+ proteinuria, meeting criteria for steroid-resistant nephrotic syndrome (SRNS).

She was readmitted for further management, including inpatient diuresis and a kidney biopsy. During this hospitalization, two doses of rituximab (375 mg/m^2^) were administered to attempt induction of remission while awaiting biopsy results.

Renal biopsy revealed focal segmental glomerulosclerosis (FSGS). Tacrolimus therapy was subsequently initiated and titrated to standard therapeutic levels of 5–10 ng/mL, and prednisone was tapered off due to lack of response. She was also started on dual proteinuria therapy with losartan 50 mg and lisinopril 20 mg to reduce proteinuria.

While on tacrolimus, lisinopril and losartan a partial response was observed, with UPCR declining from 18 mg/mg at presentation to approximately 12 mg/mg and then 10 mg/mg; however, complete remission was not achieved. Given the limited response to immunosuppressive therapy, she was referred to genetics for evaluation of a possible monogenic etiology.

Trio whole exome sequencing (WES) identified two compound heterozygous variants in the *COQ2* gene (NM_015697.7): c.683A>G (p.Asn228Ser) of paternal origin, and c.692+3A>G (splice site variant) of maternal origin. The clinical features in conjunction with the molecular findings were consistent with the diagnosis of primary CoQ10 deficiency.

Based on this diagnosis, high-dose ubiquinone (oxidized CoQ10, administered orally in a commercially available oil-based softgel formulation) was initiated at approximately 30 mg/kg/day and later increased to 40–42 mg/kg/day, corresponding to a total daily dose of about 2,000 mg. Tacrolimus was discontinued, as immunosuppressive agents are generally ineffective once a genetic cause of nephrotic syndrome is identified. Clinical improvement was evident within 2 months of CoQ10 initiation, with increased diuresis and resolution of edema and proteinuria. UPCR declined to 4.6 mg/mg within 4 months and to 1.3 mg/mg by 6 months, accompanied by normalization of serum albumin from 2.9 to 4.4 g/dL over the same period.

She remained on ubiquinone 30–40 mg/kg/day (approximately 2,000 mg/day) and lisinopril 20 mg daily. Her proteinuria stabilized (most recently UPCR 2.3), and she continued to grow and develop normally without edema. Although she demonstrated clinical improvement and a marked reduction in proteinuria, kidney function markers suggested ongoing chronic kidney disease progression. Cystatin C measured 1.95 mg/L shortly before initiation of ubiquinone supplementation and continued to rise during follow-up. Her serum creatinine stabilized between 0.89 and 0.97 mg/dL, corresponding to an estimated glomerular filtration rate (eGFR) of 57.7 mL/min/1.73 m^2^. Long-term follow-up over several years showed gradual progression of chronic kidney disease to stage 3a, consistent with irreversible renal damage secondary to longstanding CoQ10 deficiency.

Over time, her creatinine increased modestly, reaching 1.1–1.35 mg/dL. Lisinopril was later switched to losartan. Intermittent hyperkalemia (6.2 mmol/L), thought to be related to lisinopril therapy and underlying renal insufficiency, prompted the addition of the potassium binder, patiromer. Notably, her alopecia areata also improved after initiation of CoQ10 supplementation (ubiquinone). Physical examination at last follow-up showed no edema and normal neurological examination.

**Figure 1:**
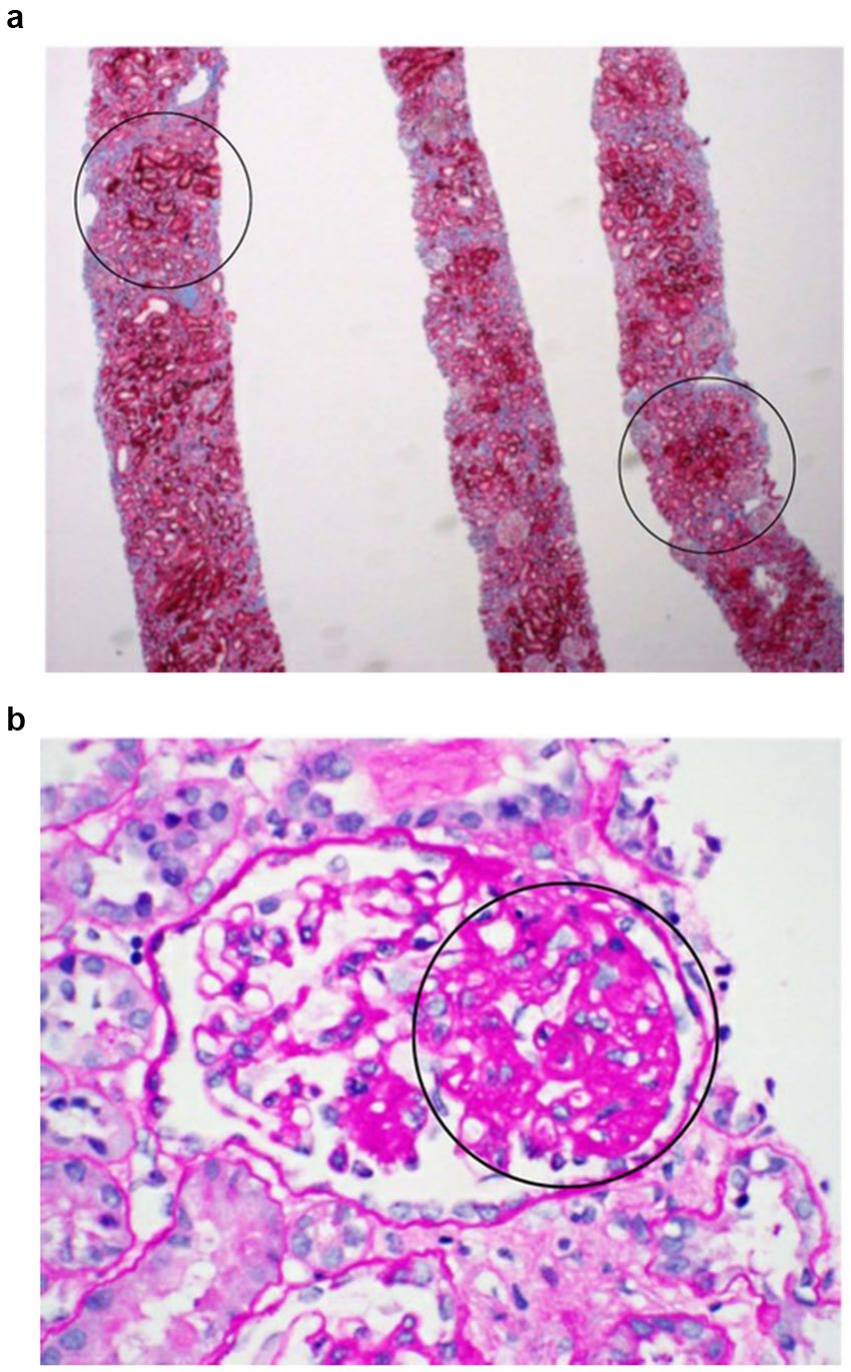
Renal biopsy: **(a)** Light microscope showed mild interstitial fibrosis with tubular atrophy. The overall cortical tubular loss is estimated to be ~20%. **(b)** Under the light microscope with PAS stain, 5 of the 40 glomeruli contained in the specimen are completely sclerotic, 17 of which are involved by segmental sclerosis. A minority of mesangial regions are mildly hypercellular.

Relevant biochemical and pathological results are summarized in the Tables 1, 2 and Figures 1a,b.

**Table 1:** Renal function test.

	During admission	Referral to genetic clinic	Start ubiquinol supplement	3 months after supplement	1 year after supplement	27 months after supplement
BUN (7–17 mg/dL)	20	18	9	16	28	25
Creatinine (0.18–0.66 mg/dL)	0.87	0.69	0.89	0.73	0.89	0.98
Albumin (3.8–5.2 g/dL)	2.3	2.4	2.9	3.2	4.4	4.2

**Table 2:** Urine protein and creatinine.

	Referral to genetic clinic	Start ubiquinol supplement	6 months after supplement	1 year after supplement	27 months after supplement
Urine protein random (mg/dL)	760	773	70	44	91
Urine creatinine random (mg/dL)	61	89	53	49	63.4
Urine protein/creatine ratio (mg/mg)	12.5	8.7	1.3	0.9	1.4

Kidney biopsy done at the time of the presentation showed some chronic changes including global sclerosis in ~10% of glomeruli, segmental sclerosis in an additional ~40% of glomeruli, and mild interstitial fibrosis (~20%). There was no significant endocapillary hypercellularity. Necrotizing lesions and cellular crescents were not seen. There were no acute glomerular proliferative changes or signs of glomerulonephritis. Under the electron microscope, extensive podocyte foot process effacement was seen which was suggestive of a primary podocytopathy. Some podocytes appeared to have an increased mitochondrial density, and a minor subset of podocyte mitochondria appeared swollen with diminished cristae. Increased mitochondrial density, extensive podocyte foot process effacement and FSGS have all been reported in patients with pathogenic variants *COQ2* (20).

### Whole exome sequencing

Whole exome sequencing (WES) showed two compound heterozygous variants in the *COQ2* gene (NM_015697.7): c.683A>G (p.Asn228Ser) and c.692+3A>G. Homozygous or compound heterozygous pathogenic variants in the *COQ2* gene have been associated with primary coenzyme Q10 deficiency-1 (COQ10D1; OMIM 607426), a rare autosomal recessive disorder involving multiple systems, which can include neurologic manifestations, steroid-resistant nephrotic syndrome (SRNS), hypertrophic cardiomyopathy (HCM), retinopathy or optic atrophy, and sensorineural hearing loss. Early treatment with high-dose oral CoQ10 supplementation can limit disease progression and reverse some manifestations (21).

The c.683A>G variant in exon 3 of the *COQ2* gene leads to a missense substitution of asparagine with serine at position 228 (p.Asn228Ser), a highly conserved amino acid. This variant was inherited from her dad. This variant has been described in multiple COQ10D1 patients with early onset SRNS as the major feature (22–26). Functional studies revealed this variant was associated with low levels of CoQ10 and reduced activities of complexes I, II, and IV of the mitochondrial respiratory chain (22). It is observed in the population (gnomAD) with a relatively low frequency of 0.011%, indicating it is unlikely benign. The University of Oklahoma lab interpreted this variant as likely pathogenic.

The c.692+3A>G variant is located at intron 3 of the *COQ2* gene. This variant was inherited from her mom. This variant has not been reported in the literature, nor has it been documented in the population database (gnomAD). The consequence of this change is not predictable without further functional studies; however, in silico analysis predicts this variant probably results in abnormal splicing. The University of Oklahoma lab classified this variant as uncertain significance.

## Discussion

Our patient was found to have two compound heterozygous variants in the *COQ2* gene: c.683A>G (p.Asn228Ser), inherited from her father, and c.692+3A>G, a novel splice site variant inherited from her mother. The c.683A>G variant is classified as likely pathogenic, while the c.692+3A>G variant, although currently classified as a variant of uncertain significance (VUS), has not been previously reported in the literature. In silico analysis using SpliceAI predicts this novel variant likely results in donor site loss (score: 0.85), indicating a potential impact on splicing. Given the patient’s clinical phenotype and the bioinformatic prediction, we suspect this novel variant is likely disease-causing.

In cases of steroid-resistant nephrotic syndrome (SRNS), renal biopsy and genetic testing are recommended components of the diagnostic workup. While awaiting genetic results, clinical guidelines recommend initiation of an ACE inhibitor or ARB (e.g., lisinopril or losartan), a calcineurin inhibitor (e.g., tacrolimus or cyclosporine), and a tapering off the corticosteroids (18).

Focal segmental glomerulosclerosis (FSGS) is the most common renal pathology associated with CoQ10 deficiency in SRNS, reported in 69% of patients with *COQ2* pathogenic variants (25 of 36 cases) (27). FSGS is classified into five histological variants: collapsing (COL), tip (TIP), cellular (CEL), perihilar (PH), and not otherwise specified (NOS) (28). These morphological features reflect podocyte injury and subsequent repair mechanisms (29). In our patient’s renal biopsy, the absence of endocapillary hypercellularity and perihilar hyalinosis supported classification as FSGS-NOS. Electron microscopy revealed podocyte abnormalities (30), including increased mitochondrial density and occasional mitochondrial swelling with loss of cristae, indicating mitochondrial and podocyte injury. Proteinuria is a hallmark of podocyte dysfunction and an early marker of kidney injury (29).

**Figure 2:**
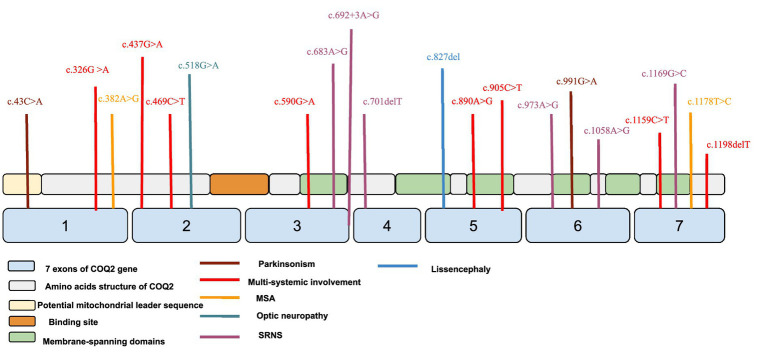
Location of *COQ2* variants reported in previous literature. The figure shows which exon each variant is located.

Notably, most cases of *COQ2*-related nephrotic syndrome reported in the literature (Supplementary Table 1) present before the age of 2 years (22, 30–34), whereas our patient had disease onset at age 9. This late-onset phenotype suggests a milder effect of the novel c.692+3A>G splice site variant on CoQ biosynthesis. Previous studies have demonstrated a genotype–phenotype correlation in *COQ2*-related disease, with severe phenotypes associated with biallelic loss-of-function variants and milder cases retaining partial CoQ production (35). Splice site mutations may result in exon 3 skipping or retention, as suggested in Figure 2. Moreover, the presence of a splice site variant also raises the possibility of future antisense oligonucleotide (ASO) therapy.

There is a known association between the degree of interstitial fibrosis and tubular atrophy and glomerular filtration rate (GFR) (36). Our patient’s biopsy showed some degree of chronic tubulointerstitial damage, which likely contributes to her persistently elevated creatinine and mildly reduced GFR despite treatment.

Pathogenic variants in *COQ2* are associated with a broad and heterogeneous spectrum of systemic manifestations. Neurological features may include fatal neonatal encephalopathy (31), infantile-onset neurodegenerative disease, and late-onset multiple system atrophy (MSA)-like syndromes characterized by autonomic dysfunction, parkinsonism, cerebellar ataxia (37), and pyramidal signs (38). In addition to steroid-resistant nephrotic syndrome (SRNS), affected individuals may present with hypertrophic cardiomyopathy (39, 40), optic atrophy or retinopathy (33, 41), and sensorineural hearing loss. Although most *COQ2*-related disorders result from biallelic pathogenic variants, certain heterozygous variants have been implicated in adult-onset MSA (37, 42). The clinical spectrum ranges from isolated nephrotic syndrome in infancy (22, 32), to early-onset multisystem disease with rapid deterioration (31), or slowly progressive adult-onset neurodegeneration (38). Notably, a recent case described a pediatric patient with isolated SRNS due to a homozygous *COQ2* variant who achieved complete remission following oral CoQ10 supplementation (43), emphasizing the importance of early genetic diagnosis and therapeutic intervention.

CoQ10 supplementation is the mainstay of treatment and should be initiated as early as possible to limit disease progression and potentially reverse manifestations (20, 30). However, severe renal or neurologic damage is often irreversible. Therefore, early genetic diagnosis is critical in children with SRNS. CoQ10 supplementation may be used in combination with ACE inhibitors in patients with persistent proteinuria (21). The protective effects of CoQ10 supplementation are believed to involve modulation of apoptotic genes (e.g., caspase-3, p53), antioxidant enzymes (e.g., PON1), and key signaling pathways such as Nrf2/HO-1 (44).

The random urine protein to creatinine ratio (UPCR) is a method of detecting and estimating the quantitative assessment of proteinuria (45). In our patient, the urine protein-to-creatinine ratio (UPCR) improved significantly from 8.7 mg/mg to 1.3 mg/mg with normalization of serum albumin levels within 6 months of CoQ10 supplementation, indicating a positive renal response. However, long-term prognosis remains uncertain, particularly regarding neurological outcomes. In a previous report, four patients with homozygous c.437G>A (p.Ser146Asn) variants and neonatal CoQ deficiency experienced only temporary improvement in nephrotic syndrome, later developing severe neurological deterioration despite early CoQ10 therapy (46). Notably, the formulation used in our patient was ubiquinone, the oxidized form of CoQ10. While several small human studies have suggested that ubiquinol, the reduced form, may achieve higher systemic CoQ10 concentrations than ubiquinone (47–49), these findings are limited and strongly influenced by the formulation matrix and solubilization methods. In particular, oil-based or softgel CoQ10 preparations are recommended to enhance absorption and bioavailability, as tablets are associated with poorer intestinal uptake (12, 50). Many of these lipid-based formulations incorporate Vitamin E to enhance the bioavailability of the lipophilic CoQ10. Although ubiquinol has been proposed to exert enhanced antioxidant or neuroprotective effects (51), conclusive clinical evidence is still lacking. Given the necessary high-dose supplementation regimen for primary CoQ10 deficiency, the inclusion of supplemental Vitamin E requires close monitoring of both the patient’s total daily intake and serum Vitamin E levels. Exceeding the tolerable upper intake level (UL) of 1,000 mg/day (1,500 IU/day) could lead to hypervitaminosis E, potentially resulting in adverse effects, most significantly an increased risk of hemorrhage.

The c.683A>G and c.692+3A>G variants identified in our patient join a growing list of *COQ2* pathogenic variants associated with isolated nephrotic syndrome, including c.701delT (23), c.973A>G (32, 52), c.1058A>G (32), and c.1169G>C (34). Given the known genotype–phenotype correlation (35), long-term outcomes following CoQ10 supplementation may depend on the extent of residual CoQ biosynthetic function. In our case, the patient showed sustained clinical improvement with high-dose CoQ10 supplementation (ubiquinone), which may have prevented further renal function deterioration and delayed or mitigated the onset of other systemic manifestations. Continued follow-up remains crucial to monitor renal function over time and to detect any emerging neurological or extrarenal involvement.

## Data Availability

The original contributions presented in the study are included in the article/Supplementary material, further inquiries can be directed to the corresponding author.
